# Stimulating effect of nanocurcumin and crocin on proliferation and pluripotency of bone marrow-derived mesenchymal stem cells

**DOI:** 10.22038/IJBMS.2024.74397.16197

**Published:** 2024

**Authors:** Nasim Sabouni, Mojgan Mohammadi, Amir Reza Boroumand, Sepideh Palizban, Jalil Tavakol Afshari

**Affiliations:** 1 Department of Immunology, Faculty of Medicine, Mashhad University of Medical Sciences, Mashhad, Iran; 2 Immunology Research Center, Mashhad University of Medical Sciences, Mashhad, Iran; 3 Neuroscience Research Center, Mashhad University of Medical Sciences, Mashhad, Iran; 4 Department of Biology, Mashhad Branch, Islamic Azad University, Mashhad, Iran

**Keywords:** Apoptosis, Cell self renewal, Cell proliferation, Mesenchymal stem cells, Nanotechnology, Plant extracts, Regenerative medicine

## Abstract

**Objective(s):**

Enhancement of proliferation, pluripotency, and self-renewal capacity as the unique features of MSCs can improve their therapeutic potential to regenerate tissues. In this context, crocin and curcumin, carotenoid compounds with outstanding medicinal properties, could be promising for cell protection and growth. This study aimed to evaluate the impact of nanocurcumin and crocin on BM-MSCs proliferation and pluripotency *in vitro*.

**Materials and Methods:**

BM-MSC were isolated from the iliac crest of SCI patients who were candidates for stem cell therapy. The effect of crocin and nanocurcumin on MSC proliferation was evaluated using MTT and PDT assay. The percentage of apoptotic MSCs was measured by flow cytometry. Furthermore, mRNA and protein expression of OCT4 and SOX2 as the proliferation and self-renewal related genes were quantified by real-time PCR and western blotting, respectively.

**Results:**

Our findings demonstrated that only low concentrations of nanocurcumin (0.3 and 0.7 µM) and crocin (2.5 5 µM) significantly affected MSCs proliferation and protected them from apoptosis. Also, crocin and nanocurcumin at low doses caused an elevation in the mRNA and protein expression levels of OCT4 and SOX2 genes. In contrast, high concentrations decreased the survival of MSCs and led to increased apoptosis compared with the untreated group.

**Conclusion:**

Our results suggest that using nanocurcumin and crocin separately in culturing MSCs can be considered proliferative agents to prepare the more advantageous tool for cell therapies. However, more *in vitro* and preclinical research is needed in this area.

## Introduction

Mesenchymal stem cells (MSCs) are multipotent cells with high self-renewal ability. They are skillful in differentiating into multiple cell lineages with mesodermal and even non-mesodermal origins. Because of their differentiation capacity into some derivatives of all three germ layers, including endoderm, mesoderm, and ectoderm, both multipotent and pluripotent terms are used for MSCs, which provide great potency in renovating a wide range of damaged tissues resulting from various diseases (1, 2). They render anti-inflammatory, anti-oxidant, anti-apoptotic, and neuroprotective activities via paracrine effects (3, 4). MSCs can be obtained from different tissues in the body. Bone marrow (BM) is one of the most appropriate sources. MSCs are easily isolated from BM and subsequently cultured and expanded under *in vitro* conditions. So, bone marrow-derived mesenchymal stem cells (BM-MSCs) have been considered candidates for regenerative medicine to treat many disorders (5).

The application of herbal medicines has a long-lasting history of curing various illnesses. In this regard during the last decades, many types of research have concentrated on the encouraging role of natural products in the proliferation, pluripotency capacity, and differentiation potential of stem cells. Curcumin and crocin, as carotenoid compounds owing to their diverse pharmacologic features, have been assumed good options for this purpose (6, 7). 

Curcumin is a yellow compound with a polyphenolic structure extracted from the rhizome of the turmeric plant *Curcuma longa* and has remarkable usage in the Middle East’s traditional medicine. It is reported to have anti-inflammatory, anti-metastatic, anti-oxidant, and neurogenic properties (8, 9). Curcumin has been shown to target stem cells by interfering with signaling pathways, which results in modifying the harmful microenvironment under the lesion area to alleviate the symptoms of the impairment (10, 11). The stimulating effect of curcumin on the proliferation, differentiation, and migration of neural progenitor cells (NPCs), neural stem cells (NSCs), and olfactory ensheathing cells (OECs) has been demonstrated in several lines of *in vitro* and *in vivo *studies. However, evaluation of this impact on MSCs seems inadequate (12). The use of curcumin has been limited because of its poor bioavailability and insolubility. Nowadays, delivery systems based on nanoparticles, including micelles, liposomes, solid lipids, etc., partly resolve these weak points (13). For example, nano-micelle-encapsulated curcumin has exhibited better pharmacokinetics in biological systems compared to the naked form (14-16). 

Crocin is the other medicinal carotenoid component obtained from saffron as the most valuable substance of *Crocus sativus* *L*. Crocin, responsible for the red color of saffron, has a hydrophilic structure (17). So far, the anti-oxidant, chemopreventive, anti-inflammatory, antidepressant, and neuroprotective effects of crocin have been revealed extensively in treating various diseases. It has been shown that crocin could regulate the activity of cancerous or normal cells at the molecular level (18-20). A few studies have investigated the induced differentiation of stem cells such as NSCs by crocin, but its role in the proliferation and pluripotency of MSCs is unexplored (21, 22).

In stem cell proliferation and differentiation, transcription factors play a critical role. Octamer-binding transcription factor 4 (OCT4) and (Sex-determining region Y)-box 2 (SOX2) are essential transcription factors for the keeping of self-regeneration in various types of stem cells (23-25). Also, these markers are associated with the proliferation and pluripotency capacity of MSCs (26-28).

The present study aimed to investigate the effect of crocin and nanomicelle of curcumin on the proliferation, apoptosis, and pluripotency of BM-MSCs *in vitro* conditions. Indeed, we explored whether crocin and nanocurcumin could separately increase BM-MSCs efficacy as cell therapeutical tools.

## Materials and Methods

***Nanocurcumin and Crocin preparation***

Nanocurcumin was purchased from Dr. Jaafari, Department of Pharmaceutical Nanotechnology, School of Pharmacy, Mashhad University of Medical Sciences, Mashhad, Iran. Curcuminoid nanomicelles were prepared using generally recognized safe (GRAS) excipients. The encapsulation of curcuminoid was performed based on Good Manufacturing Practice (GMP) rules at the Biotechnology Research Center, Bu-Ali Research Institute, Mashhad, Iran. The encapsulation efficiency of curcuminoid in nano micelle was almost 100%. The mean diameter of the nano micelle was around 10 nm, according to dynamic light scattering. Nano micelle’s curcuminoid content and size distribution remained constant for at least 24 months. The nanomicelle increases the solubility of curcuminoids to more than 100000 times. (International application No. PCT/IB2018/051370; International filing date: March 4, 2018) (14, 29). 

Crocin was purchased from Dr. Mohajeri, Department of Pharmacognosy and Biotechnology, School of Pharmacy, Mashhad University of Medical Sciences, Mashhad, Iran. It was extracted from *Crocus sativus L* using molecularly imprinted polymer solid-phase extraction by Mohajeri *et al*. based on the method represented in the Iran patent no.62215 (22, 30).

***Participants***

**Figure 1:**
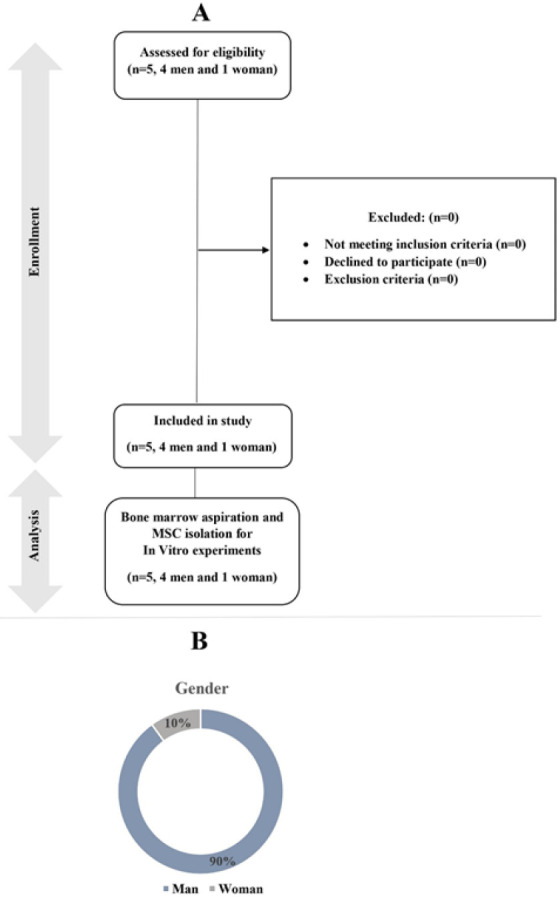
A) Consort diagram of inclusion and exclusion criteria to show the flow of participants through each stage of the study. B) Demographic chart. n: number

According to literature and available sources, also calculation using the formula: , we selected five SCI patients (four men and one woman, with a mean age of 35.4 ± 8.2 years) who were candidates for MSC therapy and met the total scores of the American Spinal Injury Association (ASIA) and SCI Functional Rating Scale of the International Association of Neurorestoratology (IANR-SCIFRS). We also evaluated them for following inclusion and exclusion criteria according to the CONSORT and demographic chart as shown in Figure 1.

***Inclusion criteria***

a- Patients who had a progressive course nonresponsive to the conventional treatments, b- Patients with no previous history of bone marrow transplantation, c- Patients with no previous history of MSC transplantation

***Exclusion criteria***

a- Patients who took prior MSC transplantation, b- Patients who took any cytotoxic drugs, c- Patients with infection, d- Patients with concomitant malignancy, e- Patients with overlap syndromes, f- Patients who were pregnant.

***Isolation and expansion of BM-MSCs***

Under local anesthesia, bone marrow aspirates were isolated from the iliac crest of the volunteers. BMs were delivered to the GMP Clean Room and diluted with (Phosphate buffered saline (PBS), pH7.4) in a 1:1 ratio. Next, MNCs (Mono Nuclear Cell) were separated using the Ficoll gradient centrifugation method (Cedarlane, Toronto, Canada).

BM-MNC were washed twice with PBS and seeded at a concentration of 10^3^ cells/cm^2^ in T25 culture flasks (SPL life science, Korea) containing minimum essential medium alpha (αMEM), 10 % fetal bovine serum (FBS; Gibco, Life Technologies), 1 % penicillin/streptomycin (100 units/ml)/ (100 μg/ml) (Gibco, Life Technologies); and maintained in a humidified, 95% air, 5% carbon dioxide (CO2), 37 °C incubator. The medium was replenished after day two, and the flasks were fed with the fresh complete αMEM (Gibco, Life Technologies). When the adherent cells reached 90% conﬂuence, they were trypsinized with 0.25% trypsin- Ethylenediaminetetraacetic acid (EDTA) (Gibco, Life Technologies) for 5 min at 37 °C. After centrifugation, detached cells were re-suspended and subcultured in T-75 flasks for subsequent cell expansion. We used passage four culture of BM-MSCs for analysis.

The study was approved by the ethics committee of Mashhad University of Medical Sciences, and written informed consent was obtained from all volunteers before conducting subsequent experiments

***Characteristics of BM-MSCs***

*Surface marker characterization of BM-MSCs by flow cytometry*

BM-MSCs were characterized according to the recommendations of ISCT (International Society for Cell Therapy). Flow cytometry analysis was done according to the manufacturer’s instructions as follows. The adherent spindle-shaped MSCs were collected from the fourth passage cultures using 0.25% trypsin-EDTA. Then, trypsin activity was neutralized by complete media containing 10% FBS, and the resultant cells were washed with staining buffer containing 90% PBS and 10% FBS. BM-MSCs were detected through the presence of Fluorescein isothiocyanate (FITC)- a cluster of differentiation (CD)90, FITC-CD73, and FITC-CD105, and the absence of FITC-conjugated CD45, FITC-CD34 (Antibodies-online, Germany). Isotype-matched antibodies were employed as controls. Finally, cell aliquots were incubated in the dark for 45 min at 4 °C and, after washing with PBS, were acquired on FACSCalibur flow cytometry (Becton Dickinson, San). FlowJo software was used for data analysis.

*In vitro osteogenic differentiation of BM-MSCs*

To recognize the osteogenic potential of BM-MSCs, cells from passage four were seeded onto 6-well plates (1×105 cells/ml) and cultured in the presence of complete αMEM. After 48 hr incubation, the medium was replaced with osteogenic differentiation media (OsteoDiff, StemMACS) composed of 2 milimolar (mM) β¬glycerol phosphate, one nanomolar (nM) dexamethasone, and 50 micromolar ( µM) ascorbate¬2¬phosphate. Finally, cells were induced under a standard situation (37 °C, 95% CO_2_) for three weeks, the culture medium was refreshed every three days, and non-stimulated cells were maintained in αMEM+FBS media alone as a control. After the end of this period, to confirm the successful osteogenic differentiation, Alizarin Red (pH 4.1) and alkaline phosphatase staining were performed to observe mineralization and detect alkaline phosphatase activity, respectively.

*In vitro adipogenic differentiation of BM-MSCs*

To evaluate the adipogenic potential of BM-MSC, after culture-expanded cells from passage four were grown to 90% confluence, BM-MSCs were trypsinized. Then, the obtained cells were plated in 6-well plates at a density of 1×10^5^ cells/ml in the presence of complete αMEM and were incubated for 48 hr. Next, the medium was discarded, and adipogenic differentiation media was replaced. Adipogenic induction medium (AdipoDiff, StemMACS), including Dulbecco’s Modified Eagle Medium (DMEM) containing 10% FBS, 10 g/ml indomethacin, 50 g/ml ascorbic acid, two mM L-glutamine, antibiotic (100 U/ml penicillin, 100 µg/ml streptomycin) and 10^7^M dexamethasone was refreshed every three days. Non-stimulated cells were kept as the control in αMEM+FBS media alone, without differentiation induction medium. After 21 days, Oil red O staining was done to observe lipid vacuoles by light microscope.

***BM-MSCs Treatment***

Harvested BM-MSCs from passage four were seeded onto 96-well plates containing αMEM+10% FBS at a final density of 5×10^3^ cells/well and kept in a humified incubator (37 °C, 95% CO_2_). When cells reached approximately 60% confluence, the previous medium was discarded. Fresh media containing nano-curcumin was added at different concentrations (0.1, 0.3, 0.7, 1.5, 3, 6, 12, 25, 50, and 100 µM), free curcumin (10 µM), micelle, only onto corresponding wells and were incubated for 48 hr. Control samples were untreated MSCs. All the processes mentioned were also carried out at different concentrations of crocin (0.625, 1.25, 2.5 and 5, 10, 25, 50, 100, and 400 µM), and control samples were untreated MSCs.

***Cell proliferation assay by MTT***

After BM-MSCs treatment according to the described procedure, the MTT (3-[4,5-dimethylthiazol-2-yl]-2,5 diphenyl tetrazolium bromide) test was used for evaluation of cell proliferation in the presence of nanocurcumin and crocin separately. First, the supernatant was removed, then 30 Microliter (µl) MTT solution (5 milligrams/ milliliter (mg/ml)) was added to each well, and then plates remained for 5 hr in a CO_2_ incubator in darkness. Media were gently removed, and 200 µl Dimethyl sulfoxide (DMSO) was added to corresponding wells to dissolve the formazan crystals. The absorbance was measured at 570 nm using an Elisa reader (31, 32).

***Calculation of population doubling time (PDT)***

PDT was calculated to measure the expansion rate of BM-MSCs, treated with (0.3 and 0.7 µM) of nano curcumin and (2.5 and 5 µM) of crocin, separately, compared to the untreated group. For this purpose, BM-MSCs from passage four were plated at a density of 5 × 10^4^ cells in each well of 6-well plates for 48 hr. Afterward**,** the cells were trypsinized and counted using trypan blue staining. Finally, PDT was calculated by the following equation: PDT = CT/PDN. In this formula, CT is the culture time, and PDN shows the population doubling number. The following formula was used to calculate PDN: 

PDN = log (N1/N0) × 3.31. In this equation, N1 is the cell number at the end of the cultivation period, and N0 is the cell number at culture initiation (33).

***Apoptosis assay***

The rate of BM-MSCs apoptosis in the presence of different concentrations of nanocurcumin and crocin was investigated by flow cytometry assay using annexin V (ANX)-FITC (Molecular Probes, Inc., BioLegend, USA) and propidium iodide (PI)-phycoerythrin (PE) ((Molecular Probes, Inc BioLegend, USA) staining according to manufacturer recommendations. Briefly, 48 hr after treatment of MSCs from passage 4 with different doses of nano-curcumin and crocin separately, all expanded cells, including treatment and control wells, were trypsinized, washed with PBS, and resuspended in 300 μl of binding buffer. Then, cells were incubated in darkness with 5 μl of Annexin V–FITC for 30 min at 37 °C. Afterward, the cells were incubated with 5 μl of PI for 15 min. Next, the cells in early and late apoptosis stages were made distinct using flow cytometric pseudocolor graphs. Early apoptotic cells were Annexin+/PI- while Annexin+/PI+ were calculated as late apoptosis, and Annexin-/PI+ were debris cells.

***Real-time PCR for OCT4 and SOX2 gene expression***

BM-MSCs from passage four were cultured at a density of 30 × 10 ^4^ cells/well in 6-well plates for 48 hr in the presence of 0.3 and 0.7 µM nanocurcumin as well as 2.5 and 5 µM crocin separately. Subsequently, total RNA was extracted (QIAGENE RNA extraction kit, Germany), and cDNA was synthesized (Yekta Tajhiz cDNA synthesis). Real-time PCR was performed using SYBR Green PCR Master mix (TaKaRa, Shiga, Japan) on the Rotor-Gene Q Machine (Qiagen, Hilden, Germany) with appropriate primers listed in Table 1. Glyceraldehyde-3-phosphate dehydrogenase (GAPDH) was used as a housekeeping gene to normalize the cycle threshold (Ct) values for the genes of interest. Based on the expression of target genes normalized to GAPDH, we calculated relative quantification ΔΔCt and the results are presented as a folded change compared to the untreated control group. The specificity of the products was determined using melting curve analysis.

***Western blotting for OCT4 and SOX2 proteins expression***

For evaluating protein expression, the BM-MSCs from passage four were treated for 48 hr in groups that exhibited a more significant effect on gene expression level, according to the results of real-time PCR. For Sox-2, the treatment groups were nanocurcumin (0.3 µM) and crocin (2.5 µM), and for Oct-4, the treatment group was nanocurcumin (0.3 µM). Also, untreated BM-MSCs were considered as the control group. 

The protein extraction was performed via the RIPA lysis buffer system (Santa Cruz, USA). The extracted proteins were analyzed for their concentrations using the Bradford method (Protein Assay Kit, Razibiotech, Iran), and supernatants were stored at -80 °C. 

The electrophoresis was run on 8% SDS-PAGE gel, and proteins were subsequently transferred onto a polyvinylidene difluoride (PVDF) membrane by a wet transfer system (Bio-Rad, USA). The membrane was blocked through incubation via TBST (Tris-buffered saline with Tween 20) buffer enriched by 3% BSA (bovine serum albumin) overnight at 4 °C. Afterwards, incubation in the presence of primary monoclonal antibody against Oct4 (1:500) or Sox2 (1:500) (Razibiotech, Iran) for two hours at 25 °C was done. Subsequently, the membrane was washed with a 1:2000 dilution of horseradish-conjugated anti-IgG secondary antibody (Razibiotech, Iran) and maintained at 25 °C for two hours. 

Using monoclonal GAPDH primary antibody at a dilution of 1:500 was considered as the internal control to normalize data. The specific bands were detected by Super Signal West Pico Chemiluminescent Substrate (Thermo Fisher Scientific, USA).

***Statistical analysis***

The results were analyzed using GraphPad Prism version 6.01 software program. One-way ANOVA followed by Dunnett’s *post hoc* test was used to determine the significant difference among groups. Also, One-sample t-test was used to analyze real-time PCR databases. The *P*-value < 0.05 was considered to be significant. All experimental procedures were repeated three times. 

## Results

***Isolation and culturing of BM-MSCs***

**Figure 2:**
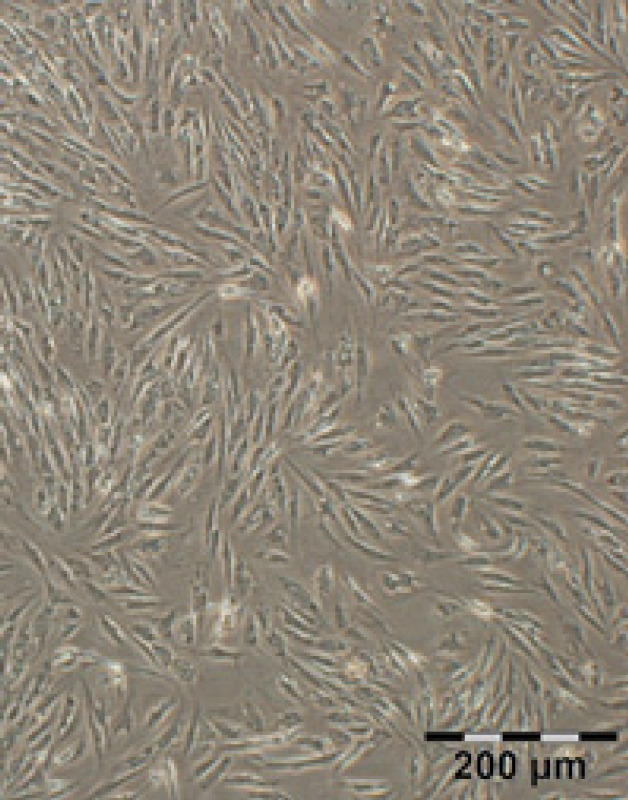
The fibroblast-like shape of BM-MSCs before differentiation at passage 4. BM-MSCs were cultured until passage four and then assessed for their morphology by light microscopy. The result indicated the fibroblast-like shape of cells, confirming the morphological characteristic of MSCs

MSC: Mesenchymal stem cell; BM: Bone marrow

After bone marrow aspiration from patients, MNC isolation by Ficoll gradient centrifugation and cell harvesting were performed successfully. Expanded BM-MSCs approximately reached 80% confluence within (10-14) days (Figure 2). MSCs with fibroblast-like morphology were displayed as a homogenous population observed microscopically. We sub-cultured cells in T-75 flasks to passage 4 for the following study assays.

***Immunophenotyping of BM-MSCs***

**Figure 3:**
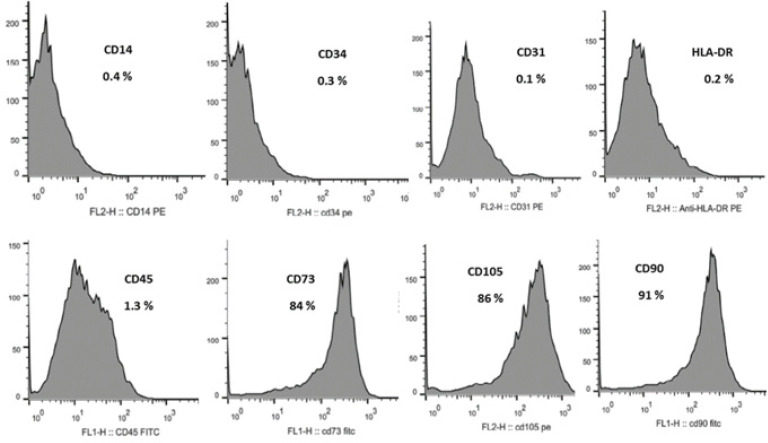
Flow cytometry analysis of superficial markers of BM-MSCs

The results of phenotype analysis by flow cytometry confirmed that the cells under investigation had characteristics of MSCs. BM-MSCs from passage four were positively expressed for CD90, CD105, and CD73 markers (>90%), whereas it was negative for non-specific markers, including CD34 and CD45 (Figure 3).

***Differentiation potential of BM-MSCs***

**Figure 4:**
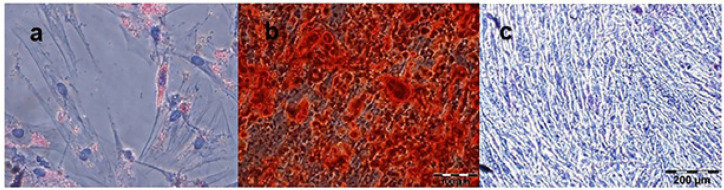
Capacity of MSCs for differentiation into adipocyte and osteocyte was established by specific-cell staining after 21 days remaining in the specialized medium

The differentiation capacity of BM-MSCs from passage four was correctly affirmed. After treating BM-MSCs with adipogenic differentiation media for three weeks, Oil red O staining revealed lipid droplets in differentiated MSCs (Figure 4, a). 

Also, after BM-MSCs treatment with osteogenic differentiation medium for 21 days, extracellular deposited calcium with bright orange-red was detected in differentiated osteocytes as bone mineralization indicator by Alizarin Red staining (Figure 4, b). Also, alkaline phosphates (ALP) activity resulting from osteogenic differentiation was determined by dark blue-violet color (Figure 4, c). Control cells did not expose any properties of differentiation following staining. 

***Effect of nanocurcumin and crocin on the BM-MSC proliferation***

**Figure 5:**
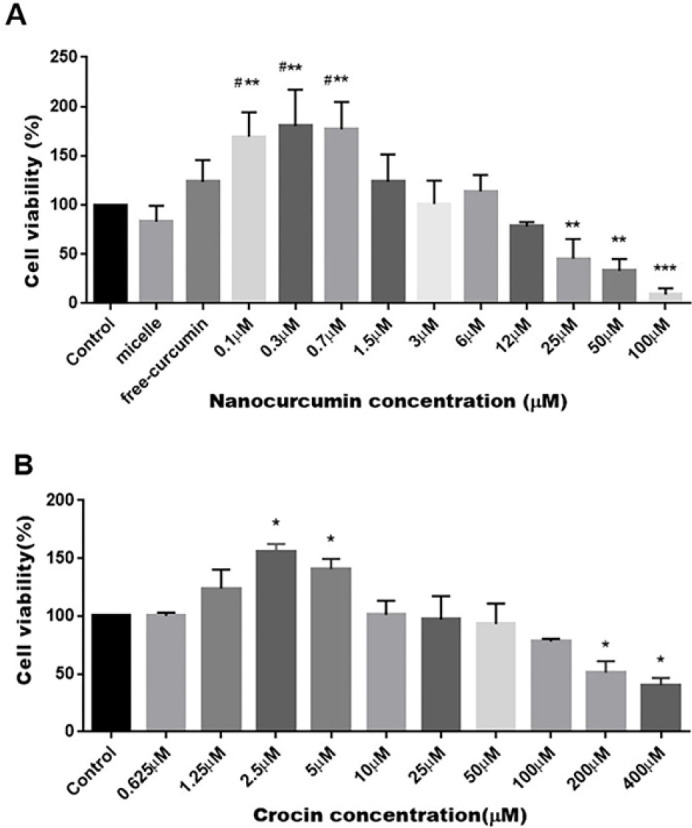
A) Rate of MSCs proliferation after incubation with increasing doses of nano-curcumin (0–100 µM) for 48 hr

After 48 hr treatment with different concentrations of nano-curcumin (0.1, 0.3, 0.7, 1.5, 3, 6, 12, 25, 50, and 100 µM), the proliferation rate of BM-MSCs was examined by MTT. The results showed that very low doses of nano-curcumin (0.1, 0.3, 0.7 µM) significantly increased MSC proliferation (*P*≤0.01) compared to untreated cells. In contrast, MSC incubation in the presence of high doses of nano curcumin led to reduction of their amplification. Free curcumin also stimulates the rate of MSC proliferation compared with the control group (Figure 5A).

Also, Proliferation of BM-MSCs was evaluated after treatment with increasing concentrations of crocin (0.625, 1.25, 2.5 and 5, 10, 15, 50,100, and 400 µM). Results showed that low doses of crocin (2.5 and 5 µM) stimulate MSC amplification significantly. According to the results, nanocurcumin and crocin indicated dose-dependent effects on BM-MSCs. The most optimal concentrations of nanocurcumin and crocin to enhance cell proliferation were (0.3 and 0.7 µM) and (2.5 and 5 µM), respectively. Un-treated MSCs were considered as negative control (Figure 5B).

***Effect of nanocurcumin and crocin on the population doubling time (PDT)***

**Figure 6:**
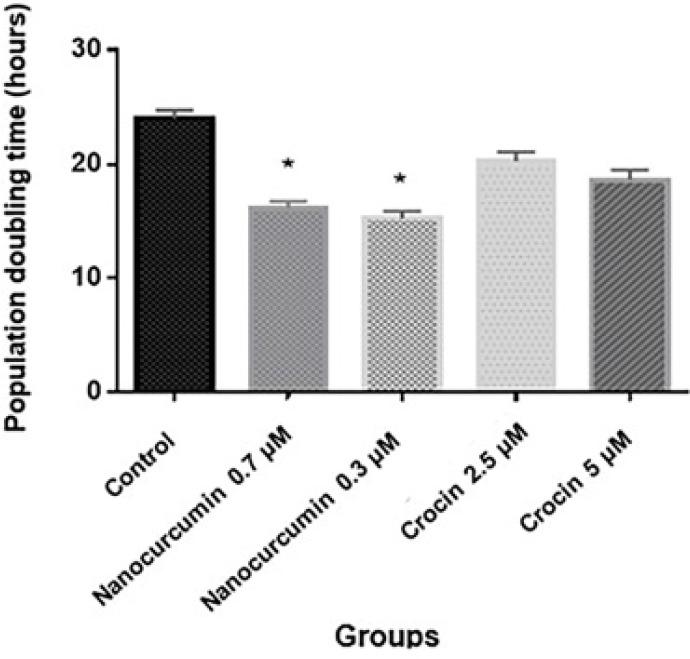
Population doubling time (PDT)

We evaluated population doubling time in the BM-MSC population by PDT test. Results indicated that PDT was decreased in all treatment groups compared with the untreated group (Figure 6). PDT value was 23.95±0.77 hr for BM-MSCs in the untreated group, while this value was calculated as 16.11±0.59 hr and 15.24±0.63 hr after treatment with 0.7 and 0.3 µM curcumin, respectively. The difference was significant (*P*<0.05). Also, PDT value was recorded at 18.60±0.85 hr and 20.21±0.83 hr after treatment with 2.5 and 5 µM crocin, respectively. The difference was not significant (*P*>0.05). The untreated group was considered a negative control.

***Effect of nanocurcumin and crocin on apoptosis of BM-MSCs***

**Figure 7:**
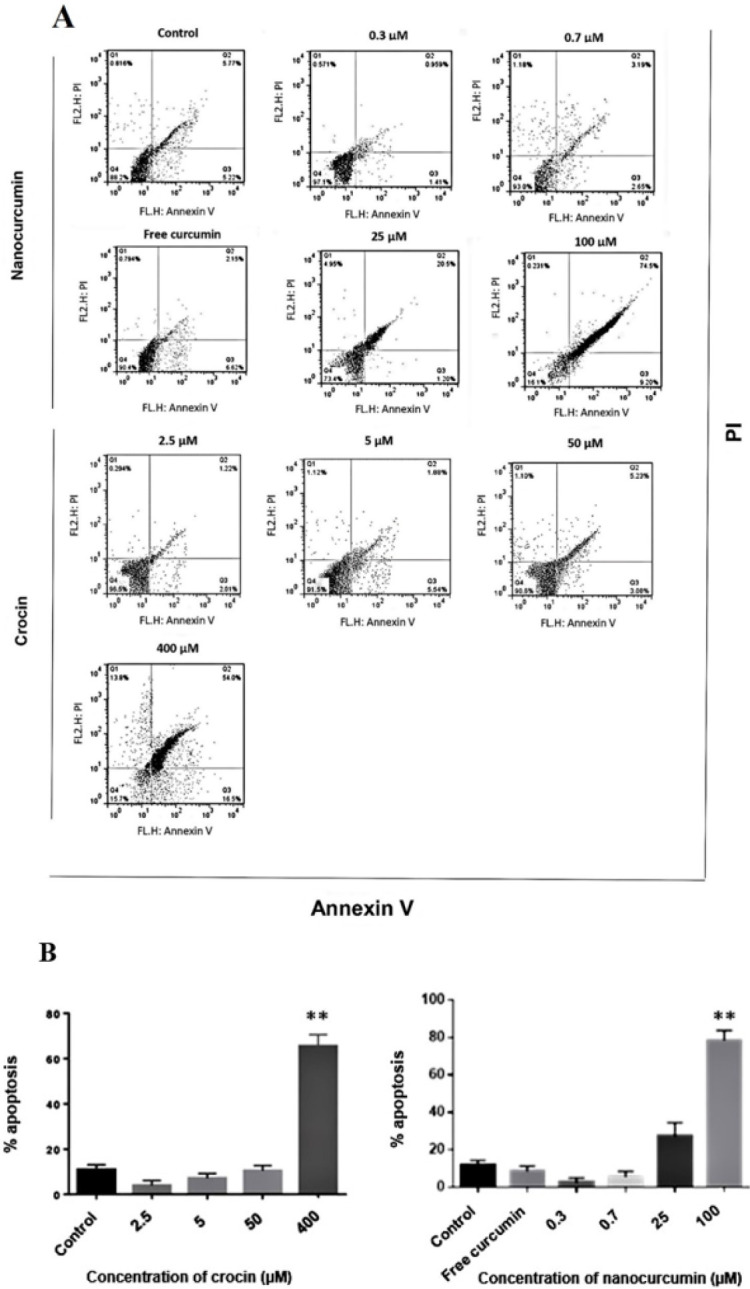
A, B) Flow cytometric analysis of apoptotic cells indicates the percentage of late apoptosis in MSCs after pretreatment with high doses of nano-curcumin and crocin is substantially increased compared with the control group (*P*≤0.01) whereas low concentrations reduce the percentage of late apoptosis in MSCs with respect to the control groups

To investigate whether the enhancement in proliferation of the MSCs may be related to the rate of apoptosis, according to the results of the MTT assay, we treated BM-MSCs with different concentrations of nano-curcumin, from low doses to high doses (0.3, 0.7, 25, and 100 µM) and with increasing concentration of crocin (2.5 and 5, 50, and 400 µM), separately. Then, the percentages of apoptotic cells using flow cytometric markers (Annexin and PI) were detected. Results showed that nano-curcumin indicates a contradictory effect on MSC apoptosis compared with MSC proliferation. Nanocurcumin at higher doses, especially 100 µM, provokes apoptosis in a dose-dependent manner (*P*≤0.01) compared with control groups (untreated cells). In contrast, at lower concentrations (0.3 and 0.7), curcumin protects MSCs from apoptosis. Results have also demonstrated that crocin poses a contrary behavior on apoptosis of MSCs when compared with MSCs proliferation, which means that crocin at higher doses, particularly 400 µM enhances apoptosis in a dose-dependent manner (*P*≤0.01) compared with control groups (untreated cells). In contrast, at lower concentrations (2.5 and 5), crocin protects MSCs from apoptosis. These findings approve a correlation between the expansion and apoptosis rate of MSCs (Figure 7).

***Effect of nanocurcumin and crocin on SOX2*** ***and OCT4 gene expression***

**Figure 8:**
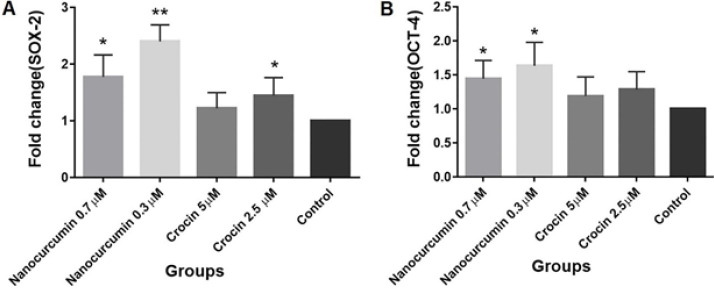
A) Relative mRNA level of SOX-2 following 48-hr treatment with different concentrations of nanocurcumin and crocin (* *P*<0.05 compared with the control group, **P<0.01 compared with the control group, n = 5). B) Relative mRNA level of OCT-4 following 48-hr treatment with different concentrations of nanocurcumin and crocin (* *P*<0.05 compared with the control group, n = 5)

We detected the expression level of SOX2 in BM-MSCs from passage four by the real-time PCR method. It was found that SOX2 mRNA increased significantly in the presence of 0.3 µM and 0.7 µM of nanocurcumin. Results showed that expression levels of SOX2 were 2.39±0.12 and 1.77±0.17 folded more than the control group (*P*=0.004, *P*=0.01), respectively, after BM-MSCs treatment with (0.3 and 0.7 µM) of nanocurcumin for 48 hr. All processes for crocin were also carried out. The results demonstrated that SOX2 mRNA increased in the presence of 2.5 and 5 µM of crocin. It was found that the expression levels of SOX2 were 1.44±0.14 and 1.21±0.12 folded more than the control group (*P*=0.03, *P*=0.1), respectively, at (2.5 and 5 µM) of crocin treatment. The difference was only significant in the presence of 2.5 µM of crocin (Figure 8A).

We also performed Real-time PCR to evaluate OCT4 expression in BM-MSCs at passage four after 48 hr of treatment with different concentrations of nanocurcumin and crocin separately. It was revealed that the expression levels of OCT4 were 1.63±0.16 and 1.47±0.12 folded more than the control group (*P*=0.01 and *P*=0.02), respectively, at (0.3 and 0.7 µM) of nanocurcumin treatment that increase was significant. All processes were also carried out for crocin. The results demonstrated that the expression levels of OCT4 were 1.28±0.11 and 1.18±0.12 folded more than the control group (*P*=0.07, *P*=0.2), respectively, at (2.5 and 5 µM) of crocin treatment but the difference was not significant. Untreated cells were considered the control group, and concentrations were selected based on the results of the MTT assay (Figure 8B).

***Effect of nanocurcumin and crocin on SOX2*** ***and OCT4 protein expression***

**Figure 9:**
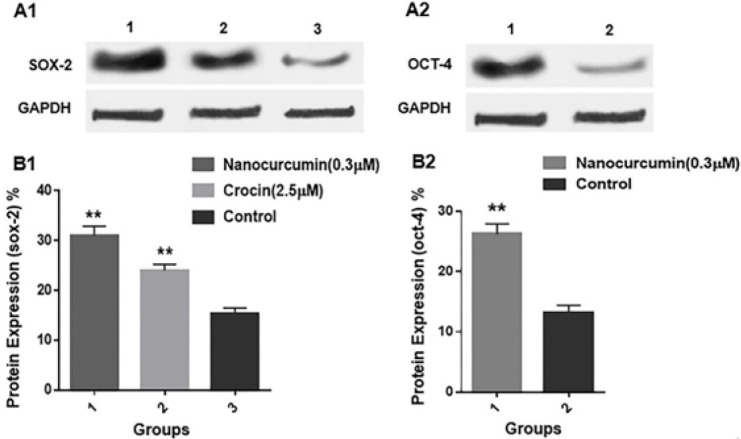
A) Western Blotting. A1) SOX-2 and GAPDH protein expression in BM-MSCs cultured in different treatment groups; lane 1= nanocurcumin (0.3 µM); lane 2= crocin (2.5 µM), lane 3= control. A2) OCT-4 and GAPDH protein expression in BM-MSCs cultured in different treatment groups; lane 1= crocin (2.5 µM), lane 2 = control. B1 and B2) Quantitative analysis of SOX-2 and OCT-4 protein was done using ImageJ software; **: *P*≤ 0.01

SOX2: (Sex-determining region Y)-box 2; GAPDH: Glyceraldehyde-3-phosphate dehydrogenase; MSC: Mesenchymal Stem Cell; BM: Bone marrow; OCT4: Octamer-binding transcription factor 4

As shown in Figure 9, western blotting results demonstrated that the Sox-2 protein contents of BMSCs in nanocurcumin (0.3 µM) and crocin (2.5 µM)-treated groups were significantly higher (*P*<0.01) than protein content in untreated BM-MSCs. Oct-4 protein expression was also increased in nanocurcumin (0.3 µM)-treated BM-MSCs compared to the control group significantly (*P*<0.01).

target tissue. Hence, stem cells and drugs could synergize each other’s effects in a carrier-carrier system (10). Since the proliferation and differentiation of MSCs. Due to pleiotropic biological activities, curcumin and crocin have received growing attention (37). Evidence has shown that these carotenoid compounds modulate signaling pathways in stem cells to direct their function in favor of improving conditions (7). Stem cells serve as the carrier for drug delivery to the free curcumin has poor bioavailability that undermines its effectiveness, nanoparticles are suggested to overcome these limitations (38). Micelle is an amphiphilic molecule in nano size capable of protecting drug particles and enhancing their water-solubility and targeted delivery (39). So, nanomicelle formulation has been introduced as a suitable carrier for enhancing curcumin functions (40). We designed current *in vitro* research to investigate the effects of crocin and micelle-encapsulated curcumin separately on the proliferation and self-renewal potential of BM-MSCs to explore the probable impact on stimulating the MSC expansion.

Our results showed that nanocurcumin exhibits a biphasic effect on bone marrow mesenchymal stem cell proliferation. MTT and PDT data illustrated that micelle-encapsulated curcumin at low concentrations increases BM-MSCs proliferation (0.3 and 0.7 µM); Flow cytometric data confirmed that low doses of nanocurcumin decrease MSC apoptosis while high concentrations (25 and 100 µM) cause stimulated cell death. In agreement with our findings, the dose-dependent effect of curcumin on stem cell viability has been documented in several studies. In a couple of studies, neural progenitor cells (NPCs) were co-cultured with curcumin, which enhanced NPCs proliferation on doses under 1 µM, whereas concentrations above 10 µM were cytotoxic (41, 42). Results from a novel study revealed that nanoformulation of curcumin improves remedial aspects of adipose-derived mesenchymal stem cells by influencing their proliferation, inflammatory cytokines, and SOD activity at low doses (43). Another study performed by Pirmoradi *et al*. in 2018 indicated the stimulating effect of curcumin on rat-derived MSc proliferation at low doses via increasing lifespan Telomerase reverse transcriptase (TERT) gene expression level responsible for reduced aging (44). An *in vitro* work in 2019 also reported that the survival of BM-MSCs in hypoxia conditions was diminished at 20 μM of curcumin (45). However, contrasting results by Li *et al*. showed that curcumin had no significant effect on the proliferation of BM-MSCs (46). Altogether, according to our latest comprehensive review, probable molecular mechanisms of curcumin to stimulate cell survival and proliferation are increasing Phosphoinositide 3-kinases (PI3Ks) and telomerase activity and activating P38 and Extracellular signal-regulated kinase (ERK) via mitogen-activated protein kinases (MAPK) pathway (47). Moreover, we indicated in the current study that crocin could exert a double-edge activity in BM-MSC survival and proliferation. Indeed, crocin played a proliferative role at low doses (2.5 and 5 µM), whereas it caused cell apoptosis at high concentrations (200 and 400 µM). So far, various research has been conducted on the impact of crocin on cancerous and normal cells to elucidate its unique therapeutic potential. Nevertheless, available data on the effect of crocin on stem cells are limited (48). Similar to our findings, recent research demonstrated that crocin and crocetin exhibit a dose-dependent effect on Adipose Tissue-derived Mesenchymal Stem Cells (AT-MSC) proliferation and its immunomodulatory properties (21). Analogously, crocin at low concentrations up to 100 μM did not have any toxicity effect on mouse BM-MSC, while higher concentrations (400 and 1000 μM) reduced cell proliferation and survival (22). Furthermore, the result of experimental work in 2014 revealed that crocin at low concentrations, including 2.5 μM, 5 μM, and 10 μM could encourage cell proliferation and anti-oxidant properties in spermatogenesis stem cells, but at 10–40 μM cause diminished cell viability (49). The speculated mechanisms involved in the inhibitory function of crocin are the interaction of this substance with telomerase-associated enzymes and interrupting the microtubule arrangement. It also can be cytoprotective mainly through rendering anti-oxidant activity and preventing protein aggregation (50-52) 

To self-renewal and pluripotency of adult stem cells, Oct4 and Sox2, are essential transcription factors. Their expression levels gradually reduce as the passage number increases. Hence, their up-regulation has been introduced to improve MSC proliferation (53, 54). In our experiment, we observed that nanocurcumin and crocin at low concentrations, which caused the increasing BM-MSCs’ proliferation, could be efficient in the up-regulation of OCT4 and SOX2 expression at mRNA level with a better impact in nano curcumin-treated cells compared with crocin-treated cells. This data was also confirmed by western blotting analysis. Results revealed the overexpression of Sox-2 and Oct-4 proteins in groups treated with low concentrations of nanocurcumin and crocin. Evidence shows that increasing OCT4 and SOX-2 expression elevated proliferation and differentiation potential in ATSCs (55). Wang *et al*. examined the proliferation of BM-MSC in Graft-versus-host disease (GVHD) patients. They stated that OCT4 and SOX2 genes are related to the self-inductance and pluripotency of stem cells (56). Nanocurcumin with dendrozum formulation at those concentrations, which reduced cell survival, down-regulated the expression of OCT4 and SOX2 in glioblastoma cells associated with neurological malignancies (57). In a study, crocin with cisplatin exhibited cytotoxic effects on ovarian cancer cells by inhibiting the expression of the SOX2 gene (58). Also, a study indicated that the fibroblastic growth factor in culture media causes increased proliferation of BM-MSCs through overexpression of OCT-4 and SOX-2 protein, which suggests the potential role of stemness-related genes in MSC proliferation (59). 

From the author’s viewpoint, to achieve satisfactory clinical outcomes of MSC therapy we first should focus on the preparation process, especially cell culture. In this regard, using proliferative and protective agents in their medium can provide more potential MSCs. The proliferation and pluripotency of MSCs are two critical characteristics of these cells in regenerating tissues, which could be stimulated by crocin and nanocurcumin based on our evidence. The efficacy of these medicinal agents is influenced by some principles, including doses, treatment period, delivery system, and source of MSC, which are optimized in current research; these results could be promising to upgrade the ameliorating capacity of MSCs.

Our study also indicated the greater efficacy of nanocurcumin with micelle formulation than free curcumin in preserving MSC survival, which suggests a positive perspective on nanotechnology-based cell culture. Nonetheless, present data can be strengthened by evaluating more genes responsible for self-renewal and pluripotency of MSCs. We also point out not evaluating probable synergy effects of crocin and nanocurcumin on MSCs in this study, which can be followed by future research.

**Table 1:** Sequence of primers used in this study

**Gene**	**Forward primer**	**Reverse Primer**
**GAPDH**	5′- AGCCGGGCATGTTCTTCAAC -3′	5′- AGGGAGCTTCACGTTCTTGTAT -3′
**SOX-2**	5′- GACAGTTACGCGCACATGAA -3′	5′- ATGTAGGTCTGCGAGCTGGT -3′
**OCT-4**	5′- CAAAGCAGAAACCCTCGTGC -3′	5′- GAACCACACTCGGACCACAT -3′

GAPDH: Glyceraldehyde-3-phosphate dehydrogenase; SOX2: (Sex-determining region Y)-box 2; OCT4: Octamer-binding transcription factor 4

**Table 2:** Result of SOX-2 protein expression (Mean values ±SD are presented)

**Nanocurcumin (03 µM)** (Mean±SD)	**Crocin (2.5 µM)** (Mean±SD)	**Control** (Mean±SD)
30.96±1.938	24.00±1.195	15.34±1.095

SOX2: (Sex-determining region Y)-box 2

**Table 3:** Result of OCT-4 protein expression (Mean values ±SD are presented)

**Nanocurcumin (03 µM)** (Mean±SD)	**Control** (Mean±SD)
26.22±1.718	13.23±1.165

OCT4: Octamer-binding transcription factor 4

Histogram plots showed that BM-MSCs are positive (more than 80%) for the expression of CD73, CD105, and CD90, whereas negative (less than 2%) for CD34, CD31, CD14, CD45, and HLA-DR. Data were indicated on 10000 events

MSC: Mesenchymal stem cell; BM: Bone marrow

After differentiation of BM-MSCs toward adipocytes, lipid vacuoles were detected using staining with the Oil Red O method (a). Mineralization potential of BM-MSCs was detected by the appearance of calcium deposits following Alizarin Red staining (b) and alkaline phosphatase staining (c).

MSC: Mesenchymal stem cell; BM: Bone marrow

Data were analyzed using One-way ANOVA followed by Dunnett’s *post hoc* test. Nano-curcumin at very low concentrations (0.1, 0.3, and 0.7 µM) stimulates MSC proliferation significantly compared with control groups (untreated cells). However, higher concentrations (25–100) indicate an inhibitory effect on MSC expansion compared to control groups

* Significance vs un-treated cells. ** Significance vs un-treated cells (*P*≤0.01), *** Significance vs un-treated cells (*P*≤0.001) # significance vs free curcumin. (*P*≤0.05), Mean values ±SD are presented, (n=5).

B) Rate of MSCs proliferation after incubation with increasing doses of crocin (0-400 µM) for 48 hr

Cell expansion was evaluated using a 3-(4,5-dimethylthiazol-2-yl)-2,5- diphenyl tetrazolium bromide (MTT) assay. * significance vs un-treated cells (control), The values are expressed as mean± standard deviations (SD) of triplicate determinations (n=5). Significantly defined as *P*≤0.05. *

MSC: Mesenchymal stem cell

Data were analyzed using One-way ANOVA followed by Dunnett’s *post hoc* test; *Indicates significant PDT of 0.3 and 0.7 µM nanocurcumin-treated cells compared with that of untreated cells (*P*<0.05).

**significantly different from 0 µM (untreated cell). Data are indicated as mean values ±SD. Flow Cytometry data analysis was done using FlowJo Software, and then data (percentages of apoptotic cells) were analyzed using One-way ANOVA followed by Dunnett’s *post hoc* test.

MSC: Mesenchymal stem cell

Data were analyzed using One-sample t-test

mRNA: messenger ribonucleic acid; SOX2: (Sex-determining region Y)-box 2; OCT4: Octamer-binding transcription factor 4

## Conclusion

MSC characteristics can be varied concerning their morphology, proliferation rate, and self-renewal capacity, which determine its therapeutic potential and are influenced by cell culture conditions. Our study suggests that crocin (2.5 and 5 µM) and nanocurcumin (0.3 and 0.7 µM) as complementary factors in the culture of BM-MSCs can stimulate their proliferation and self-renewal, so help to prepare a better cell product for patients who undergo MSCs therapies. This research could be the basis for encouraging future investigations on combination therapy using stem cells and medicinal plants. However, further preclinical and clinical studies are needed to develop a well-established protocol.

## References

[1] Vasanthan J, Gurusamy N, Rajasingh S, Sigamani V, Kirankumar S, Thomas EL (2021). Role of human mesenchymal stem cells in regenerative therapy. Cells.

[2] Farkhad NK, Mahmoudi A, Mahdipour E (2021). How similar are human mesenchymal stem cells derived from different origins? a review of comparative studies. Curr Stem Cell Res Ther.

[3] Babu S, Krishnan M, Panneerselvam A, Chinnaiyan M (2023). A comprehensive review on therapeutic application of mesenchymal stem cells in neuroregeneration. Life Sci.

[4] Biglari N, Mehdizadeh A, Mastanabad MV, Gharaeikhezri MH, Afrakoti LGMP, Pourbala H (2023). Application of mesenchymal stem cells (MSCs) in neurodegenerative disorders: history, findings, and prospective challenges. Pathol Res Pract.

[5] Chu D-T, Phuong TNT, Tien NLB, Tran DK, Thanh VV, Quang TL (2020). An update on the progress of isolation, culture, storage, and clinical application of human bone marrow mesenchymal stem/stromal cells. Int J Mol Sci.

[6] Saud B, Malla R, Shrestha K (2019). A review on the effect of plant extract on mesenchymal stem cell proliferation and differentiation. Stem Cells Int.

[7] Milani A, Basirnejad M, Shahbazi S, Bolhassani A Carotenoids: biochemistry, pharmacology and treatment. Br J Pharmacol.

[8] Urošević M, Nikolić L, Gajić I, Nikolić V, Dinić A, Miljković V (2022). Curcumin: Biological activities and modern pharmaceutical forms. Antibiotics (Basel).

[9] Shah M, Murad W, Mubin S, Ullah O, Rehman NU, Rahman MH (2022). Multiple health benefits of curcumin and its therapeutic potential. Environ Sci Pollut Res Int.

[10] Sharifi S, Zununi Vahed S, Ahmadian E, Maleki Dizaj S, Abedi A, Hosseiniyan Khatibi SM Stem cell therapy: Curcumin does the trick. Phytother Res.

[11] Kabir M, Rahman M, Akter R, Behl T, Kaushik D, Mittal V (2021). Potential role of curcumin and its nanoformulations to treat various types of cancers. Biomolecules.

[12] Heidari S, Mahdiani S, Hashemi M, Kalalinia F (2020). Recent advances in neurogenic and neuroprotective effects of curcumin through the induction of neural stem cells. Biotechnol Appl Biochem.

[13] Wang H, Zhou Y, Sun Q, Zhou C, Hu S, Lenahan C (2021). Update on nanoparticle-based drug delivery system for anti-inflammatory treatment. Front Bioeng Biotechnol.

[14] Hatamipour M, Sahebkar A, Alavizadeh SH, Dorri M, Jaafari MR (2019). Novel nanomicelle formulation to enhance bioavailability and stability of curcuminoids. Iran J Basic Med Sci.

[15] Na Q, Xiyou D, Ji J, Zhai G (2021). A review of stimuli-responsive polymeric micelles for tumor-targeted delivery of curcumin. Drug Dev Ind Pharm.

[16] Trigo-Gutierrez JK, Vega-Chacón Y, Soares AB, Mima EGdO (2021). Antimicrobial activity of curcumin in nanoformulations: a comprehensive review. Int J Mol Sci.

[17] Song Y-n, Wang Y, Zheng Y-h, Liu T-l, Zhang C (2021). Crocins: A comprehensive review of structural characteristics, pharmacokinetics and therapeutic effects. Fitoterapia.

[18] Hashemzaei M, Mamoulakis C, Tsarouhas K, Georgiadis G, Lazopoulos G, Tsatsakis A (2020). Crocin: A fighter against inflammation and pain. Food Chem Toxicol.

[19] Poursamimi J, Shariati-Sarabi Z, Tavakkol-Afshari J, Mohajeri SA, Mohammadi M (2020). Crocus sativus (Saffron): An immunoregulatory factor in the autoimmune and non-autoimmune diseases. Iran J Allergy Asthma Immunol.

[20] Kermanshahi S, Ghanavati G, Abbasi-Mesrabadi M, Gholami M, Ulloa L, Motaghinejad M (2020). Novel neuroprotective potential of crocin in neurodegenerative disorders: An illustrated mechanistic review. Neurochem Res.

[21] Yousefi F, Arab FL, Rastin M, Tabasi NS, Nikkhah K, Mahmoudi M (2021). Comparative assessment of immunomodulatory, proliferative, and anti-oxidant activities of crocin and crocetin on mesenchymal stem cells. J Cell Biochem.

[22] Kalalinia F, Ghasim H, Farzad SA, Pishavar E, Ramezani M, Hashemi M (2018). Comparison of the effect of crocin and crocetin, two major compounds extracted from saffron, on osteogenic differentiation of mesenchymal stem cells. Life Sci.

[23] Wang G, Zhou H, Gu Z, Gao Q, Shen G (2018). Oct4 promotes cancer cell proliferation and migration and leads to poor prognosis associated with the survivin/STAT3 pathway in hepatocellular carcinoma. Oncol Rep.

[24] Ghourichaee SS, Powell EM, Leach JB (2017). Enhancement of human neural stem cell self-renewal in 3D hypoxic culture. Biotechnol Bioeng.

[25] Strebinger D, Deluz C, Friman ET, Govindan S, Alber AB, Suter DM (2019). Endogenous fluctuations of OCT 4 and SOX 2 bias pluripotent cell fate decisions. Mol Syst Biol.

[26] Zhang Z-Y, Hou Y-P, Zou X-Y, Xing X-Y, Ju G-Q, Zhong L (2020). Oct-4 enhanced the therapeutic effects of mesenchymal stem cell-derived extracellular vesicles in acute kidney injury. Kidney Blood Press Res.

[27] Bharti D, Shivakumar SB, Park J-K, Ullah I, Subbarao RB, Park J-S (2018). Comparative analysis of human Wharton’s jelly mesenchymal stem cells derived from different parts of the same umbilical cord. Cell Tissue Res.

[28] Wu Q, Fang T, Lang H, Chen M, Shi P, Pang X (2017). Comparison of the proliferation, migration and angiogenic properties of human amniotic epithelial and mesenchymal stem cells and their effects on endothelial cells. Int J Mol Med.

[29] Rahimi HR, Mohammadpour AH, Dastani M, Jaafari MR, Abnous K, Mobarhan MG (2016). The effect of nano-curcumin on HbA1c, fasting blood glucose, and lipid profile in diabetic subjects: a randomized clinical trial. Avicenna J Phytomed.

[30] Mohajeri SA, Hosseinzadeh H, Keyhanfar F, Aghamohammadian J (2010). Extraction of crocin from saffron (Crocus sativus) using molecularly imprinted polymer solid-phase extraction. J Sep Sci.

